# Supra-pubic Cystotomy

**Published:** 1892-06

**Authors:** L. A. Grizzard

**Affiliations:** Abilene, Texas


For Daniel's Texas Medical Journal.

### SUPRA-PUBIC CYSTOTOMY.

BY L. A. GRIZZARD, M. D., ABILENE, TEXAS.

ON THE 10th of March last, assisted by Drs. Alexander and Pipkin, I did a supra-pubic cystotomy upon Luke A——, a boy seven years of age, removing a calculus, oxalate lime, beautiful specimen, about the size and shape of a large chestnut, weighing —— grs. The patient was thoroughly washed with soap and water, and all the parts in opposition to the hypogastric region thoroughly washed in a bi-chloride solution, anesthetized, rectum inflated, bladder moderately distended with an aseptic fluid, towels, hands and instruments rendered aseptic, an incision through the skin over linea alba about three inches in length was made, extending slightly below the pubic summit, dissecting carefully the superimposed tissues, checking hemorrhage until the bladder was reached, when, with a tenaculum, the bladder was siezed, while two silk ligatues, one on either side, were passed into the walls of the bladder, for the purpose of holding the organ in position during the remainder of the operation.

An incision in the bladder was made, beginning upon a level with the pubic bone, and extending upward an inch, possibly more. The solution removed, finger introduced, stone located and removed without difficulty. The bladder was thoroughly washed out, and the wound irrigated with solution of bi-chloride. A Nelaton's soft catheter was now introduced into the bladder down to the prostate gland, and where it passed from the abdominal cavity stitched with silk to the abdominal walls to retain it in position, the other end passed over the pubis and between the thighs, and into a vessel.

At the upper and lower angle of the cystic wound, catgut sutures were introduced through all the tissues save the mucous (?) or lining membrane of the bladder, the same ligature, including in its exit the entire abdominal wall, thereby approximating, or bringing in juxtaposition, the cystic and abdominal wall, and, I believe, by so doing minimize the danger from extravasation of urine, the principal source of mortality from this high operation.

The entire cystic and abdominal wounds, except the space occupied by the drainage tubes, were closed with interrupted suture.

Iodoform and bi-chloride gauze and absorbent cotton, held in place by spica bandage, constituted the dressing.

Gave a slight re-action, temperature 102; his condition was excellent until the third day, when bronchitis supervened, and for a few days his condition was serious, when, happily, the clouds disappeared, and he was satisfactorily journeying the road to recovery, until the sixth day from the operation, in a fit of anger, prompted, doubtless, by the Adamic spirit so prevalent in all human beings (save doctors), he violently jerked from the wound the drainage tube, thus re-opening the wound that had so kindly healed.

In twelve hours fever arose, and for four or five days he tremblingly (I did most of the trembling) hovered near the crumbling brink that separates time from eternity. For several days, during granulation, the urine trickled from the abdominal wound like a small spring from the mountain's bosom, and yet no extravasation of urine took place. Why? I believe it was because the bladder was attached to the abdominal wall, and an exudation of lymph acted as a barrier to extravasation.

With all his concurrent evils and mishaps, in three weeks from the date of operation he was well.

Without the unfortunate bronchitis and untimely drainage tube removal, his recovery, I believe, would have been unmolested, and without an untoward event.

